# Pathological conditions re-shape physiological Tregs into pathological Tregs

**DOI:** 10.1186/s41038-015-0001-0

**Published:** 2015-05-28

**Authors:** William Y Yang, Ying Shao, Jahaira Lopez-Pastrana, Jietang Mai, Hong Wang, Xiao-feng Yang

**Affiliations:** 1 Centers for Metabolic Disease Research, Cardiovascular Research, and Thrombosis Research, Department of Pharmacology, Temple University School of Medicine, MERB 1059, 3500 North Broad Street, Philadelphia, PA 19140 USA; 2 Department of Microbiology and Immunology, Temple University School of Medicine, Philadelphia, PA 19140 USA

**Keywords:** Regulatory T cells, Immune suppression, Epigenetic mechanisms, Histone modifications, Metabolic cardiovascular diseases

## Abstract

CD4^+^FOXP3^+^ regulatory T cells (Tregs) are a subset of CD4 T cells that play an essential role in maintaining peripheral immune tolerance, controlling acute and chronic inflammation, allergy, autoimmune diseases, and anti-cancer immune responses. Over the past 20 years, a significant progress has been made since Tregs were first characterized in 1995. Many concepts and principles regarding Tregs generation, phenotypic features, subsets (tTregs, pTregs, iTregs, and iTreg35), tissue specificity (central Tregs, effector Tregs, and tissue resident Tregs), homeostasis (highly dynamic and apoptotic), regulation of Tregs by receptors for PAMPs and DAMPs, Treg plasticity (re-differentiation to other CD4 T helper cell subsets, Th1, Th2, Tfh, and Th17), and epigenetic regulation of Tregs phenotypes and functions have been innovated. In this concise review, we want to briefly analyze these eight new progresses in the study of Tregs. We have also proposed for the first time a novel concept that “physiological Tregs” have been re-shaped into “pathological Tregs” in various pathological environments. Continuing of the improvement in our understanding on this important cellular component about the immune tolerance and immune suppression would lead to the future development of novel therapeutics approaches for acute and chronic inflammatory diseases, allergy, allogeneic transplantation-related immunity, sepsis, autoimmune diseases, and cancers.

## Introduction

CD4^+^FOXP3^+^ regulatory T cells are classified as a subpopulation of CD4 T cells specialized in the suppression of immunopathogenic responses from the host immune system against self or foreign antigens and dangerous substances [1,2]. The suppressive function of Tregs in the maintenance of self-tolerance and prevention of the development of autoimmune and chronic inflammatory diseases is mediated by different mechanisms such as Tregs killing of target cells [3], modulation of target cell signaling via cell-cell contact and/or secretion of anti-inflammatory cytokines such as interleukin-10 (IL-10), IL-35 [4,5], and transforming growth factor β (TGF-β) [4,6] as well as modulation of target cells by exosome-carried microRNAs. Currently, several experimental systems are commercially available that simplify the identification, isolation, and characterization of Tregs using fluorescent-conjugated antibodies for CD4, CD25, FOXP3, CD127, cytotoxic T-lymphocyte associated molecule-4 (CTLA-4), glucocorticoid-induced tumor necrosis factor (TNF) receptor (GITR), CD39, and CD45RA [7].

Originally termed suppressor T cells, the recognition of regulatory T cells as a cellular mechanism for immune tolerance resulted from experiments performed in the 1960s and 1970s by Gerson and Kondo, which described the induction of suppressor T cells capable of downregulation of antigen-specific T cell responses [8]. Due to the lack of known molecular markers, research on suppressor T cells ceased. However, in 1995, Sagakuchi et al*.* identified CD25 as a surface phenotypic marker for suppressive CD4 cells in mice [9]. Since then, suppressive T cells have been called regulatory T cells (Tregs). Since the first characterization of Tregs in 1995 [9,10], one of the major milestones in Tregs studies was the identification of FOXP3. FOXP3 is a member of the forkhead/winged-helix family of transcription factors, which acts as a “master regulator” (for multiple pathways) for the development and suppressive function of Tregs [11-13]. The significance of FOXP3 gene was identified by its mutations that cause fatal autoimmune diseases in early life, which is now termed immunodysregulation, polyendocrinopathy, enteropathy, and X-linked (IPEX) syndrome in mice and humans. Since the discovery of the FOXP3 gene, its role and modification have been one of the potential topics in translational medicine field due to the essential function of FOXP3 in maintaining immune tolerance and homeostasis. Similar to T-bet (a T-box gene encoded transcription factor) [14], GATA3 (a trans-acting T cell-specific transcription factor) [15], and RORγt (a RAR-related nuclear orphan receptor family member) [16] identified as the subset-specific transcription factors for the terminal differentiation of type 1 CD4 T helper cells (Th1), Th2, and Th17, respectively, identification of FOXP3 as a Treg-specific transcription factor was a logic approach for the establishment of Tregs as a terminally differentiated and lineage committed subset of CD4 T cells.

**Figure 1:**
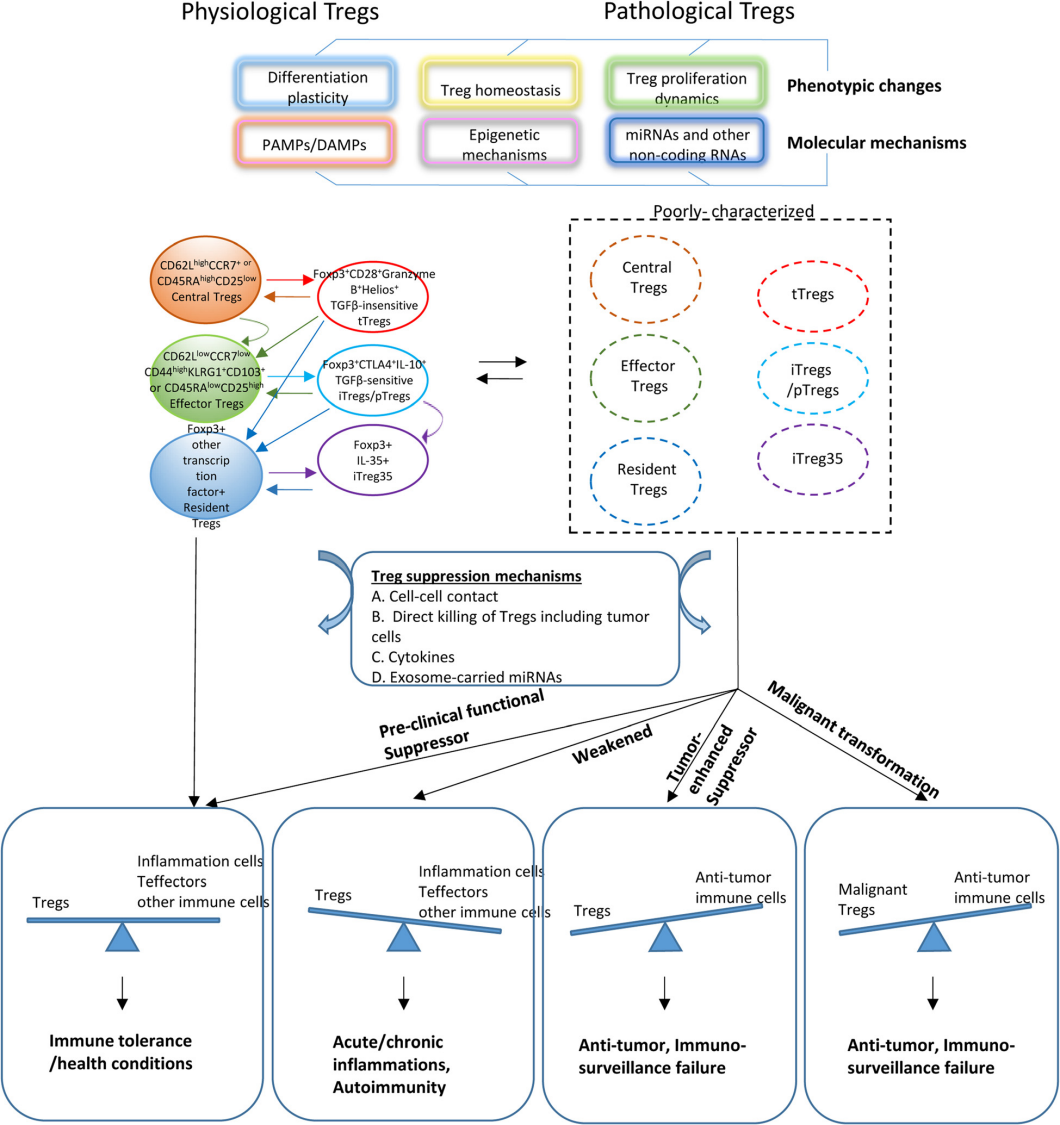
Our novel working model has been proposed: “Pathological conditions re-shape physiological Tregs into pathological Tregs”.

Due to its significant roles in controlling inflammation, innate and adaptive immune responses, and immune tolerance, many unsolved questions remain to be the focus of investigations [17], such as 1) whether Tregs have to be generated in the central lymphoid organ like thymus or Tregs can be “converted” from non-Tregs in peripheral tissues [18,19]. Recently, a special population of Tregs was found in injured muscle, which is distinct from other Treg population and potentiates muscle repair. This report not only revealed a new function of Tregs in modulating tissue repair but also proposed a new pathway for Treg generation [20]; 2) whether there are tissue-resident Tregs, in other words, whether tissue physiological environment modulates Tregs’ genotypes and phenotypes; 3) how do Tregs maintain their own homeostasis. In 2008, we analyzed more than 90 factors involved in regulating homeostasis of Tregs, suggesting that the repertoires of Tregs are undergoing constant adjustment [21]and finally three mechanistic issues were addressed; 4) whether Tregs have functional receptors to sense pathogen-associated molecular patterns (PAMPs) or danger signal-associated molecular patterns (DAMPs) and participate in inflammation [2,22,23]; 5) whether Tregs are a non-changeable lineage committed subset or have differentiation plasticity; and 6) whether epigenetic mechanisms [19] regulate Tregs’ response to changes in physiological tissue environments and pathological conditions such as inflammation. Of note, Treg homeostatic changes have been reported in various diseases [24-66] as shown in Table 1, in which the PMIDs of the papers/reviews are listed. Based on the significant progresses, we have proposed for the first time a novel concept that “physiological Tregs” have been modulated into “pathological Tregs” in various pathological environments. As shown in our novel working model in Figure 1, both physiological and pathological Tregs may undergo changes in differentiation/subset plasticity, homeostasis (survival/death), and proliferation dynamics via molecular mechanisms including pathogen (danger)-associated molecular pattern (PAMPs/DAMPs) receptor signaling, epigenetic mechanisms, microRNAs, and other noncoding RNAs. The issue of how pathological conditions re-shape the subsets of physiological Tregs remains to be poorly addressed. Pathological Tregs can have four different functional status including a) functional suppressors, b) weakened suppressors, c) tumor-enhanced suppressors, and d) malignant Tregs. Improvement of our understanding on these important issues would eventually lead to the future development of Tregs-based novel therapeutics for treating acute and chronic inflammatory diseases, allergy, allogeneic transplantation-related immunity, sepsis, autoimmune diseases, and cancers. For more historical details on the research progress of Tregs, one may refer to others’ excellent reviews as well as ours [2,13,16,18,19,21].

**Table 1:** **Treg homeostatic changes have been found in various diseases**

**Pathological conditions**	**Major phenotypes**	**PMIDs**	**Reference #**
Atherosclerosis coronary disease (CAD)	Tregs in chronic stable angina patients →	20539016	[23]
	Tregs in patients with ST-elevation acute myocardial infarction ↑		
	Tregs in patients with non-ST-elevation acute coronary syndrome patients ↓		
	Ratio of CD4^+^CD2S^+^Foxp3^+^/CD4^+^ T cells in patients with acute coronary syndrome ↓	18294918	[24]
	CD4^+^CD2S^+^Foxp3^+^ Tregs in patients with unstable CAD ↓	17512253	[25]
End stage kidney disease	Tregs sensitivity to Fas-mediated apoptosis	20429423	[26]
Type II diabetes			
	Ratio of CD4^+^CD2S^high^ Treg/Th17 ↓	21964948	[27]
	Ratio of CD4^+^CD2S^high^ Treg/Th1 ↓	21169542	[28]
	Peripheral induced CD4^+^Foxp3^+^Helios^−^ Tregs ↓		
	Bcl-2/Bax ratio in CD4^+^CD2S^high^ Tregs ↓		
Obesity-linked insulin resistance	Natural Tregs↓	21911743	[29]
	Adaptive Tregs in visceral adipose tissue ↑		
Obesity	Circulating C04^+^CD25÷CD127-Foxp3^+^ Tregs ↓ and inversely correlated with body weight	23592653	[30]
	Visceral adipose Tregs ↓	21298111	[31]
Allergy	Individuals may develop allergy (Th2 predominant) or recovery (Tr1 predominant) depending on the balance between allergen-specific Th2 and Tr1	15173208, 14987885	[32,33]
		12704370	[34]
	CD4^+^CD25^+^ Tregs inhibit TH1 and TH2, cytokine production in atopic patients		
SLE (lupus)	CD4^+^CD2S^+^, CD4^+^CD69^+^ and CD4^+^CD2S^high^ Tregs ↓	14599852	[35]
	The frequency →, and function of CD4^+^CD25^+^ cells ↓, CD4^+^Foxp3^+^ →	16890406	[36]
	The ratio and number of CD4^+^CD25^high^Foxp3^+^ nTregs ↓	17670847	[37]
	The ratio of CD4^+^IL-1G÷ iTregs, but the number →		
Rheumatoid arthritis	CD4^+^CD2s^bright^ Tregs cell in joint fluid ↑	15807863,	[38]
	CD4^+^CD2s^bright^ Tregs cell in peripheral blood ↓	15225369, 16571607	[39,40]
	Function of CD4^+^CD25^+^Tregs ↓	15280421	[41]
Severe juvenile idiopathic arthritis	CD4^+^CD25^bright^ ↓	15128835	[42]
Sepsis	Ratio of circulating CD4.CD25÷CD45RO^+^CD69^−^ Tregs/CD4^+^CD25— Teffectorst ↑	12847405, 15640650	[43,44]
		18946659	[45]
	Increased CD4^+^CD25^+^CD127^−^Foxp3^+^ Tregs contribute to lymphocyte anergy Percentage of CD4^+^CD25^+^ Treg ↑	15640650	[44]
	Resistance of Treg to apoptosis processes	11292647, 15817707	[46,47]
Injury	CD4^+^CD25^+^ Treg function ↑ after burn injury	16365414	[48]
	CD3^+^CD4^+^CD2S^high^CDl27^low^Foxp3^+^ ↑ in patients with acute lung injury	19770521	[49]
Graft rejection	The frequencies and proportion of CD4^+^CD2S^+^Foxp3^+^ Tregs → in allograft acceptors and rejecters, but the Foxp3 expression levels in Tregs in acceptor patients are 50% higher than rejecter patients	19109145	[50]
	Cotransfer of purified CD4^+^CD2S^+^ Tregs along with the CD4^+^CD25^−^ T cells significantly delay graft versus host disease	11390438	[51]
	Inducible Tregs prolong allograft survival without newly formed innate Tregs entering the periphery	14707064	[52]
	TCR^+^CD4^−^CD8^−^ Tregs mediate acceptance of skin allografts by inducing the deletion of alloreactive CD8^+^ T cells	10888927	[53]
	FoxP3^+^ Treg/CD3^+^ T cell ratio positively correlated with graft function at 2 years after transplantation	18495961	[54]
Cancer	CD4^+^CD25^high^ Tregs ↑ in circulating and tumor infiltrating lymphocytes (TILs) in patients with epithelial malignancies, inhibiting the proliferation of conventional T cells and IFN-y production	11466340	[55]
	CD4^+^CD25^high^ Tregs with positive lL.10/TGF-p/CTLA-4 ↑ in peripheral blood, lymph nodes and tumor tissue in patients with pancreatic and breast cancer	12193750	[56]
	IL-lO-producing CD4^+^CD2S^high^ Tregs ↑ in PB, TILs, draining LNs, and ascites fluid in gastro-esophageal cancers, which were strongly associated to disease stage	14555512, 15734494, 16328385, 12942579	[57-60]
	CD4^+^CD25^+^Foxp3^+^ Tregs ↑ in PB, malignant ascites, tumoral tissue, and draining INs in patients with ovarian cancer patients	15322536	[61]
	Circulating CD4^+^CD25^high^ Tregs ↑ in chronic lymphocytic leukemia (CLI), B cell-derived non-Hodgkin lymphomas (B-NHL5)	15914560, 16403912, 17047079	[62-64]
	CD4^+^CD25^high^CD45^−^RA^−^CD69^−^CD45RO^+^CD9S^+^ Tregs ↑ in acute myeloid leukemia and present higher apoptosis and proliferation	16313258	[65]

↑: significant increase, ↓: significant decrease, →: no change.

## Review

### tTregs, pTregs, iTregs, and iTreg35

FOXP3^+^CD28^+^Granzyme B^+^TGFβ-insensitive naturally occurring thymus-generated Tregs (tTregs or nTregs) [67] are characterized by the expression of CD4, and transcriptional factor FOXP3 and high expression of CD25 [68]. Initially identified for their co-expression of CD4 and CD25 cell surface markers, in subsequent reports tTregs have been recognized by other surface markers such as CD103, CD62L, lymphocyte activation gene 3 protein (LAG 3), C-C chemokine receptor type 5 (CCR5), neurophilin-1 [69-71], the activation antigens glucocorticoid-induced tumor necrosis factor receptor (TNFR) family related gene (GITR), and cytotoxic T-lymphocyte-associated protein 4 (CTLA-4) (also known as CD152), as well as the lack of certain cell surface markers such as CD127 (the α chain of the IL-7 receptor) [72].Of note, Helios, a member of Ikaros transcription factor family is proposed to be a marker of tTregs [73], which can be used to distinguish tTregs from iTregs [67]. Fully matured FOXP3^+^ tTregs exit the thymus and migrate to the secondary lymphoid organs where they suppress the proliferation of tissue-specific autoimmune T cells, restrain their differentiation into type 1 T helper cells (Th1), Th2, and Th17 lineages *in vivo* [74], and inhibit polyclonal T cell activation and T-effector cell trafficking [67]. They recognize specific self-antigens and prevent autoimmunity by the inhibition of pathogenic lymphocytes. In addition to adaptive immune cells such as T cells and B cells, tTregs also inhibit the function of innate immune cells including antigen-presenting cells such as macrophages and dendritic cells (DCs) by modulating expression of MARCHI and CD83 on macrophages and DCs [67,74].

So far, the modes of how do tTregs suppress effector cells have been identified: inhibition of cytokine production, prevention of cytotoxic T cells proliferation, and inactivation of antigen-presenting cell function. In addition, Tregs contribute to effector T cells apoptosis via three mechanisms including Fas-FasL-mediated killing, suppressive cytokine-induced impairment [6,75,76], and microRNA transmitted in exosomes [77]. As we reviewed [18], the role of tTregs in suppressing chronic inflammation has been clearly demonstrated in experimental atherosclerosis model in 2006 by Ait-Oufellaet et al., which showed an increase in atherosclerotic lesion size and vulnerability in proatherogenic apolipoprotein E deficient (ApoE−/−) mice after peripheral Tregs were depleted [18,78].

FOXP3^+^CTLA4^+^IL-10^+^TGFβ-sensitive adaptive, inducible, or peripheral Tregs (iTregs/aTregs/pTregs) [79,80] are induced by T cell antigen receptor (TCR) ligation (antigen-specific Tregs) [67] and TGFβ stimulation in periphery from CD4^+^CD25^−^ T cell precursors [21,79], which acquire the upregulation of CD25 (interleukin-2 receptor α chain (IL-2Rα)). Inducible Tregs are developed from naïve CD4 T cells in the lymphoid tissues in response to specific antigens in the presence of cytokines such as transforming growth factor-β1 (TGF-β1), interleukin-10 (IL-10), and IL-4, while in the absence of pro-inflammatory cytokines such as interferon-γ (IFN-γ), IL-1, IL-6, and IL-12. This antigen presentation in the absence of danger signals is referred as tolerogenic, which is essential for the suppression of undesired immune reactivity against non-harmful materials such as airborne particles, commensal bacteria, and foods. In addition, iTregs depend on IL-2 for development and survival as previously reported [21,81-83], which also explains why iTregs highly express CD25 and probably other IL-2 receptor components. Furthermore, iTregs may be able to redirect macrophage differentiation toward an anti-inflammatory cytokine-producing type 2 macrophage phenotype (M2) rather than pro-inflammatory type 1 macrophages (M1 phenotype) [9].

Different subsets of iTregs have been reported including T regulatory cell type 1 (Tr1) and T helper cell type 3 (Th3) [84]. Tr1 cells are CD25^−^FOXP3^−^CD49b^+^LAG-3^+^T cells characterized by the secretion of large amounts of IL-10, some IL-5 and IFN-γ with or without TGF-β, IL-2, or IL-4 [85,86]. Tr1 cells control the activation of naïve and memory T cells *in vivo* and *in vitro* and also suppress the Th1 and Th2 immune responses to pathogens, tumors, and alloantigen-expressed transplanted tissues [87]. The capacity of DCs to induce T cell proliferation is strongly reduced by the supernatant of activated Tr1 [88], suggesting that Tr1 suppression is mediated by secreted cytokines. Th3 cells have been shown to produce high amounts of TGF-β when induced by oral tolerance in mucosal tissue in an antigen-specific manner [74]. In addition, CD4^+^LAP^+^ (latency-associated peptide) Tregs have been identified recently as the third iTregs subtype whose suppression is mediated by TGF-β in immune diseases including experimental autoimmune encephalitis (EAE), type I diabetes mellitus (T1DM), systemic lupus erythematosus (SLE), collagen-induced arthritis, type II diabetes, and atherosclerosis in mice [74,89].

Most recently, a new suppressive cytokine IL-35 [6,75,76] has defined a new IL-35-producing iTregs subset, iTreg35 [75], and a subset of IL-35-producing B regulatory cells (Breg) [90,91]. These induced cell subsets are probably responsive to acute and chronic inflammation stimuli since we previously reported that IL-35 is a new category of suppressive cytokine termed responsive cytokine in contrast to the house-keeping suppressive cytokine TGF-β [4].

### Tissue-resident Tregs

Similar or parallel to the classification of tTregs, pTregs, iTregs, and iTreg35, it has been proposed that Tregs can also be classified into three new subsets, central Tregs, effector Tregs, and tissue-resident Tregs [17]. Of note, every new classification of cell subsets reflects the improvement of our understanding on the features of the cells. Central Tregs, also termed as resting Tregs or naïve Tregs, make up the majority of Tregs in the circulation and secondary lymphoid organs. Central Tregs have a phenotype of CD62L^high^CCR7^+^ (C-C chemokine receptor type 7) or CD45RA^high^CD25^low^. Effector (memory) Tregs constitute a minor fraction of Tregs in the circulation and secondary lymphoid organs, which have a phenotype of CD62L^low^CCR7^low^CD44^high^KLRG1^+^ (killer cell lectin-like receptor subfamily G member1-positive) CD103^+^ or CD45RA^low^CD25^high^. Tissue-resident Tregs specify those Tregs that have a long-term residence in non-lymphoid tissues. So far, four types of tissue-resident Tregs have been identified: 1) skin/lung Tregs are specified by two transcription factors FOXP3 and T-bet. The trafficking of skin/lung Tregs are controlled by CXCR3 (receptor for the C-X-C chemokine CXCL9, CXCL10, and CXCL11) and CCR4 (chemokine (C-C motif) receptor 4). The homeostatic mediators for skin/lung Tregs include CD40L, IFN-ɤ, IL-27, and IL-7; 2) gut Tregs are specified by two transcription factors, FOXP3 and Signal transducer and activator of transcription 3 (STAT3). The trafficking of gut Tregs are controlled by CCR6. The homeostatic mediators for gut Tregs include short-chain fatty acid (SCFA), IL-10, IL-6, and IL-1; 3) germinal center Tregs are specified by two transcription factors, FOXP3 and BCL-6. The trafficking of germinal center Tregs are controlled by CXCR5. The homeostatic mediators for germinal center Tregs are still unknown; and 4) adipose tissue Tregs [92] are specified by two transcription factors, FOXP3 and peroxisome proliferator-activated receptor gamma (PPARɤ). The chemokine receptors for trafficking of adipose tissue Tregs are unknown. The homeostatic mediators for adipose tissue Tregs include lipids and long-chain fatty acid (LCFA) [17]. In the near future, we will see more characterizations of tissue-resident Tregs. Identification of tissue-resident Tregs suggests that Tregs express additional transcription factors, chemokine receptors, and receptors for homeostatic mediators in order to respond to the signals of tissue physiological environments.

### Highly dynamic and apoptotic populations of Tregs

Our previous reports showed that CD25^+/high^ Tregs are a cell population that are “hungry” for IL-2 for survival and undergo apoptosis easily by upregulating pro-apoptotic protein Bax [81,93] and downregulating anti-apoptotic cytosolic protein translationally controlled tumor protein (TCTP) [82,83,94]. Postulating from our updated understanding on CD25^low^ central Tregs and CD25^high^ effector Tregs, our results suggest that CD25^low^ central Tregs are less likely to undergo acute coronary syndrome- [95], end stage renal disease- [27], and chronic inflammation/infection-related [17], IL-2 decrease-triggered Treg apoptosis [96-98] than CD25^high^ effector Tregs. Based on our results and others’ reports, we proposed that the Tregs apoptotic pathways are novel therapeutic targets for the future development of novel therapy in treating inflammation, autoimmune disease, transplantation-related diseases, allergy, and cancers [21]. Our findings were confirmed by recent excellent reports on high rates of Tregs apoptosis. These reports show that Treg-specific transcription factor FOXP3 is a pro-apoptotic protein [99]; and that mitochondrial anti-apoptosis-regulatory pathway protein Mcl-1 is critical for Tregs survival and niche-filling capacity [100]. However, future work is still needed to determine the apoptotic rates for each subset of Tregs.

High apoptotic rates of Tregs raises the question of how these Tregs maintain their population sizes. Using a special technique to follow up the Thy-1 expressing recent thymic cell emigrants (RTE), investigators found that approximately 30% of CD4^+^CD25^+^FOXP3^+^ Tregs express the markers associated with RTE. Following thymectomy, the numbers of cells expressing these markers fell by 80% within 30 days. In addition, although only ~5% of CD4 single-positive thymocytes express FOXP3 within 24 h after intrathymic injection of fluorescence dye fluorescein isothiocyanate (FITC), more than 30% of the labeled CD4^+^RTE are FOXP3^+^, suggesting that some RTE may acquire FOXP3 expression in the periphery. Thus, some RTE may acquire FOXP3 rapidly after emigration from thymus. Tregs are dividing rapidly with apparent half-lives of ~18 days and ~7 days for the CD4^+^CD25^+^FOXP3^+^ and CD4^+^CD25^−^FOXP3^+^ subsets, respectively. The apparent slower turnover of CD4^+^CD25^+^FOXP3^+^ cells is a result of CD4^+^CD25^+^FOXP3^+^ to CD4^+^CD25^−^FOXP3^+^ conversion, with no loss of regulatory function. Therefore, the data suggested that Tregs in adults are relatively short-lived and Tregs numbers are maintained by rapid cell division and continuous replenishment from the thymus [101].

### Pathogen-associated molecular pattern receptors on Tregs

After a long time of extensive research, it has been widely accepted that the host innate immune system is equipped with a set of receptors to recognize PAMPs derived from viruses, bacteria, other invasive microorganisms and environmental stimuli or metabolite-related DAMPs [2,22,23]. So far, four types of receptors for PAMPs and DAMPs have been identified; Toll-like receptors (TLRs, 13 members), nucleotide-binding oligomerization domain (NOD) leucine-rich-repeat containing receptors (NLRs, 18 members), C-type lectin receptors (5 members), and retinoic acid-inducible gene I protein (RIG-I) helicase receptors (2 members) [102]. An important question remains whether Tregs are equipped with these sets of receptors for PAMPs and DAMPs. It was reported that stimulation of human Tregs with a mixture of TLR2 ligands Pam2CSK4, Pam3CSK4, and FSL-1 can result in a reversal of suppression on anti-CD3/anti-CD28 stimulated responder T cells. However, the gastric mucosa of the infected TLR2 knock-out mice showed a lower mRNA expression of FOXP3, IL-10, and IL-17A, but a higher expression of IFN-γ compared to the gastric mRNA expression in infected wild-type mice [103]. In addition, TLR5 and TLR8 also modulate the suppressive activity of naturally occurring CD4^+^CD25^high^ Tregs. The suppressive capacity of Tregs is counter-regulated by TLR ligands indirectly via antigen-presenting cells (APCs) or responder T cells or directly [104]. Similarly, TLR4 stimulation of T effector cells and Tregs co-culture leads to a more pronounced T effector cell activation [105]. TLR ligand lipopolysaccharide (LPS) stimulation of TLR4 on Tregs, similar to T cell antigen receptor ligation, can enhance Tregs suppression on neutrophils [106]. It is obvious that this field is still in the early stage. Further studies are needed to determine the roles of the receptors for PAMPs and DAMPs in regulating Tregs homeostasis and suppressive function.

### Tregs plasticity and FOXP3 expression regulated by other transcription factors

Current understandings suggest that naïve CD4 T helper cells can differentiate/polarize into at least six different terminally differentiated/lineage-committed subsets. 1) Naïve CD4 T helper cells can differentiate into type 1 T helper cell subset (Th1) with the expression of Th1-specific transcription factor T-bet in the presence of IL-12 and IFN-γ. 2) Naïve CD4 T cells can differentiate into Th2 subset with the expression of Th2-specific transcription factor GATA3 in the presence of IL-4. 3) Naïve CD4 T cells can differentiate into Th17 subset with the expression of Th17-specific transcription factor RORγt in the presence of IL-6 and TGF-β. 4) Naïve CD4 T cells can differentiate into iTregs subset with the expression of Treg-specific transcription factor FOXP3 in the presence of TGF-β, retinoic acid, and IL-2. 5) Naïve CD4 T cells can also differentiate into follicular T helper cells (Tfh) subset in the presence of IL-21. Naïve CD4^+^ T cells and Th2 can differentiate into Th9 cells in the presence of TGF-β [107,108].

Among all these CD4 subsets, Th1 and Th2 are relatively stable, but iTregs and Th17 cells can readily switch to other T helper subsets under certain cytokine stimulations. In the presence of B cells and CD40-CD40L interaction, iTregs can switch to follicular T helper (Tfh) cells mediated by transcription factor B-cell lymphoma/leukemia 6 (BCL-6) [109]. In addition, iTregs can also switch to IL-17-producing cells (Th17) mediated by transcription factor STAT3 [109] after stimulation with IL-6 and IL-21. Moreover, iTregs can also convert to Th2 cells mediated by transcription factor Irf4 [109]. Furthermore, iTregs can switch to Th1 cells mediated by T-bet [109]. Finally, Th17 can convert into IFN-γ-producing Th1 cells or IL-4-producing Th2 cells when stimulated by IL-12 or IL-4, respectively. Th2 cells convert to IL-9-producing cells in response to TGF-β stimulation [108]. This conversion of terminally differentiated lineage committed subset to other terminally differentiated lineage committed subsets is termed “plasticity”. Several factors participate in the regulation of T cell conversion including extrinsic and intrinsic ones. The extrinsic factors include accessory immune cells, innate receptors for PAMPs and DAMPs, cytokine microenvironment including the cytokines in polarizing other T helper subsets, cytokine receptor regulation, nutrient availability, and metabolic pathways including long-chain fatty acids and short-chain fatty acids for adipose tissue Tregs. The intrinsic factors are cell cycle and phenotype stability, microRNA mediated control of T cell phenotype, transcription factor dosing and dominance, and epigenetic modifications [109].

In fact, numerous transcription factors have been identified in reshaping Tregs development, function, and homeostasis. These include the first group of transcription factors functional in regulating FOXP3 expression and the second group of transcription factors in forming a complex with FOXP3. Some of the factors are overlapped in these two groups. The first group includes nuclear factor of activated T cells (NFAT), c-Rel (nuclear factor kappa-light-chain-enhancer of activated B cells (NF-kB)), activator protein 1 (AP-1), Nr4a (a subfamily of the orphan nuclear receptors), and signal transducer and activator of transcription 5 (STAT5), and FOXP3. In addition to the regulation of FOXP3 gene expression, recent reports indicate that FOXP3 itself is able to form complexes with a number of co-factors to execute cooperative effects during their interaction [110]. The second group of FOXP3 co-factors is composed of as many as 11 sequence specific transcription factors including NFATc2, runt-related transcription factor 1 (RUNX1; also known as acute myeloid leukemia 1 protein (AML1) or core-binding factor subunit alpha-2 (CBFA2)), B-cell lymphoma/leukemia 11B (BCL11b), Foxp1, Foxp4, GATA-3, STAT3, Ikaros (Ikzf1; a lymphoid transcription factor LyF-1), Aiolos (Ikzf3; the Ikaros, Aiolos and Helios are three family members of the hematopoietic specific transcription factors involved in the regulation of lymphocyte development), Ets (E26 transformation-specific family of transcription factors), and Cnot3 (CCR4-NOT transcription complex, subunit 3). The majority of FOXP3 binding sites within the genome lack an identifiable forkhead-binding motif in Tregs, which suggests that a large number of FOXP3 co-factors facilitate the binding of FOXP3 to a given site [111-113]. Since some of these transcription factors, if not all, are also inflammation-regulatory transcription factors [114], it suggests that the inflammation and pathological conditions can regulate the plasticity of Tregs and other T cells. In support of this argument, the expression of Treg-specific transcription factor FOXP3 requires demethylation of FOXP3 promoter [115] whereas proatherogenic stimuli like oxidized low-density lipoprotein (oxLDL) induces increased methylation of FOXP3 promoter and decreased FOXP3 expression [116].

### Epigenetic regulation and microRNA regulation of Tregs

Under inflammatory stimulations, the suppressive function of Tregs is decreased and TGF-β-induced Tregs development is attenuated by an epigenetic manner [117]. It suggests that epigenetic regulation of Tregs function, development, and homeostasis is patho-physiologically relevant. In correlation with this finding, Tregs suppression is also reported to be attenuated in autoimmune type 1 diabetes, in which epigenetics is one of the pathological mechanisms involved [118]. What is epigenetics? Epigenetics refers to heritable changes that occur in gene expression without modification in the DNA sequence of the genome. These epigenetics mechanisms, which briefly include DNA methylation/demethylation, histones modifications, and micro-RNAs (miRNAs) are the principal mechanisms involved in regulating chromosomal organization, chromosomal remodeling and then gene expression via different dynamic levels. More specifically, it has been demonstrated that epigenetics mechanisms play a critical role in regulating FOXP3 expression and lead to further regulations in Tregs functions and homeostasis [119-121]. Emerging epigenetics therapies are providing new therapeutic agents for the control of various diseases [13,16,18,21,81-83,115,122].

The term epigenetics was first introduced by Conrad Waddington in 1942 [123]. Epigenetics [123] integrates organism genotypes by the influence and response of environmental stimuli on their phenotype, which can take place in chromosomal DNA or in the proteins linked with the chromosomal DNA such as histones. In recent years, many epigenetic proteins have been investigated in laboratories and in clinic, while inhibitor development for modification enzymes is the frontier for drug discovery. So far, epigenetic modifications have been grouped into four main categories: DNA methylation, histone modification including histone methylation/demethylation, histone acetylation/deacetylation, histone phosphorylation, histone SUMOylation [124], small and long noncoding RNAs, as well as chromatin remodeling [124,125].

Noncoding RNAs (ncRNAs) are a type of functional RNA molecule, which are not translated into proteins. More functional groups of ncRNAs have been categorized by the following: four short noncoding RNAs (17–31 base pairs (bp)) (microRNAs (miRNAs), Piwi-interacting RNAs (piRNAs), small interfering RNAs (siRNAs), and transcription initiation RNAs), mid-size noncoding RNAs (<200 bp) (small nucleolar RNAs, promoter-associated small RNAs (PASRs), TSS-associated RNAs (TSSa-RNAs), and promoter upstream transcripts (PROMPTs)), long noncoding RNAs (lncRNAs, >200 bp), and its subgroups such as long intergenic noncoding RNAs (lincRNAs), enhancer RNAs (eRNAs), transcribed ultraconserved regions (T-UCRs) and other lncRNAs [126]. It has been widely shown that ncRNAs not only regulate gene expression at the transcriptional and post-transcriptional levels but also play a role in the control of epigenetic pathways [127]. Of note, we recently published a comprehensive review on epigenetics enzymes as new therapeutic targets for Treg-based therapy, which one may refer to for the details [19].

## Conclusions

The roles of Tregs in inhibiting inflammation, autoimmune diseases, allergy, transplantation, and modulating anti-cancer immune responses have been well established. Due to extensive studies, several new concepts and principles regarding Tregs in physiological and pathological status have emerged, which are concisely discussed in this brief review. Continuation in improving our understanding on the molecular mechanisms underlying Tregs development, homeostasis, and functions will eventually lead to the future development of novel Tregs-based therapeutics to treat inflammation, chronic cardiovascular diseases, allergy, allogeneic transplantation-related immune responses, sepsis, autoimmune diseases, and cancers.
